# Distinct serum GDNF coupling with brain structural and functional changes underlies cognitive status in Parkinson's disease

**DOI:** 10.1111/cns.14461

**Published:** 2023-09-17

**Authors:** Chuanxi Tang, Ruiao Sun, Ke Xue, Mengying Wang, Sijie Liang, Piniel Alphayo Kambey, Mingyu Shi, Changyu Wu, Gang Chen, Dianshuai Gao

**Affiliations:** ^1^ Department of Neurobiology, Xuzhou Key Laboratory of Neurobiology Xuzhou Medical University Xuzhou Jiangsu China; ^2^ Department of Epidemiology and Biostatistics, School of Public Health Peking University Health Science Center Beijing China; ^3^ Department of Rehabilitation The Affiliated Hospital of Xuzhou Medical University Xuzhou Jiangsu China; ^4^ School of Medical Imaging Xuzhou Medical University Xuzhou Jiangsu China; ^5^ Department of Neurology Shuyang Hospital of Traditional Chinese Medicine Suqian Jiangsu China

**Keywords:** cognitive dysfunction, cortical thickness, degree centrality, fMRI, GDNF, Parkinson's disease

## Abstract

**Aim:**

Aberrations in brain connections are implicated in the pathogenesis of Parkinson's disease (PD). We previously demonstrated that Glial cell‐derived neurotrophic factor (GDNF) reduction is associated with cognition decline. Nonetheless, it is elusive if the pattern of brain topological connectivity differed across PD with divergent serum GDNF levels, and the accompanying profile of cognitive deficits has yet to be determined.

**Methods:**

We collected data on the participants' cognition, demographics, and serum GDNF levels. Participants underwent 3.0T magnetic resonance imaging, and we assessed the degree centrality, brain network topology, and cortical thickness of the healthy control (HC) (*n* = 25), PD‐high‐GDNF (*n* = 19), and PD‐low‐GDNF (*n* = 19) groups using graph‐theoretic measures of resting‐state functional MRI to reveal how much brain connectivity varies and its clinical correlates, as well as to determine factors predicting the cognitive status in PD.

**Results:**

The results show different network properties between groups. Degree centrality abnormalities were found in the right inferior frontal gyrus and right parietal lobe postcentral gyrus, linked with cognition scores. The two aberrant clusters serve as a potentially powerful signal for determining whether a patient has PD and the patient's cognition level after integrating with GDNF, duration, and dopamine dosage. Moreover, we found a significant positive relationship between the thickness of the left caudal middle frontal lobe and a plethora of cognitive domains. Further discriminant analysis revealed that the cortical thickness of this region could distinguish PD patients from healthy controls. The mental state evaluation will also be more precise when paired with GDNF and duration.

**Conclusion:**

Our findings reveal that the topological features of brain networks and cortical thickness are altered in PD patients with cognitive deficits. The above change, accompanied by the serum GDNF, may have merit as a diagnosis marker for PD and, arguably, cognition status.

## Graphical abstract

This study was based on a clinical case–control study to investigate whether serum GDNF level is related to the occurrence of PD‐CI and which domain of cognitive impairment is most closely related. Imaging markers were further explored within different levels of serum GDNF PD patients. Degree centrality abnormalities were found. The two aberrant clusters serve as a potentially powerful signal for determining whether a patient has PD or not, as well as cognition level after integrating with GDNF, duration, and dopamine dosage. Moreover, we found a significant positive relationship between the thickness of the left caudal middle frontal lobe and a plethora of cognitive domains. The cognitive state evaluation would also be more precise when paired with GDNF and duration.

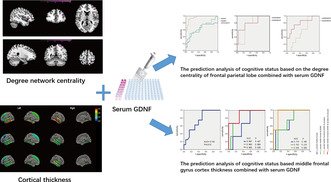

## 1 INTRODUCTION

Parkinson's disease (PD) is a multifactorial degenerative disease that affects over 6 million people worldwide.^1^, ^2^ Alongside the characteristic physical motor symptoms, other non‐motor symptoms, such as loss of smell, cognitive dysfunction, constipation, and sleep disorders, have gradually been recognized, suggesting that the clinical presentation of PD is multifaceted.^3^ An increased emphasis on cognitive impairment has recently appeared in the early stages for up to 42.5% of PD patients,^4^ and the diagnostic time point of the principal canonical criteria of tremor, rigidity, and bradykinesia has brought about significant research progress.^5^ The main features of the cognitive dysfunction of PD are deficits in executive function and visuospatial function.^6^ In addition, PD patients with early cognitive impairment are at a higher risk of developing dementia in a shorter period.^7^ Diagnosis of PD with early cognitive decline has been viewed as a substantial contributor to disability and poor quality of life, considerable nursing difficulty, and family and social burden.

Because the pathophysiological process of cognitive impairment in PD has not been fully understood, the constant discovery of numerous biomarkers deserves to be explored despite a lack of standard diagnostic techniques.^8^ It is noteworthy that method advances, such as the availability of neuroimaging techniques and the application of network science, have resulted in a rise in the accuracy and confidence of multimodal combination testing.^9^ Resting‐state functional MRI has shown the ability to examine spontaneous brain function.^10^ As the analytical method of fMRI advances, newer and more powerful algebra‐topological methods can express changes in brain network topological features at the system level.^11^ Several studies have confirmed that PD with mild cognitive impairment has aberrant network activation in the default network mode.^12^

Moreover, combining imaging studies also suggests that genetic characteristics, brain Aβ‐amyloid depositions, and serum or cerebrospinal fluid biomarkers play a distinct role in cognitive deterioration.^13^, ^14^ In other words, neuroimaging techniques could be integrated into multi‐aspect biomarker exploration methods to improve the diagnostic sensitivity and specificity of clinical diseases or symptoms, such as PD with cognition impairment (PD‐CI). Most importantly, imaging can also provide unique ideas and insights into the mechanism of PD‐CI from the standpoint of a neural network.

Glial cell‐derived neurotrophic factor (GDNF) potently promotes dopaminergic neuron survival. It presents significantly lower blood levels in PD patients.^15^, ^16^ GDNF levels, in particular, are lower in PD‐CI subjects and are correlated with a range of cognitive scales.^16^, ^17^ As a result of autopsy studies, there is evidence that GDNF is reduced in the central nervous system of PD patients.^18^, ^19^ Our team has been concerned about the effects of GDNF on dopaminergic neuron (DAN) survival and terminal dopamine transmission, and we demonstrated that depletion of GDNF impairs dopamine transmission in prefrontal terminals.^20^ With the recent advance in single‐cell RNA sequencing technology, it has been found that DAN subpopulations exist and show different brain region connectivity.^21^ These studies indicate that changes in GDNF level and brain region connectivity suggest possible involvement in regulating PD cognition. Moreover, circulating GDNF levels, which mirror GDNF alterations in the brain, show great potential as a marker for clinical PD diagnosis. Crucially, previous studies have left unresolved the issue of whether PD patients with circulating GDNF differences also had changes in their brain networks and whether the two variables might contribute to PD‐CI.

Most studies have considered classifying cognitive impairment to compare and validate the indicators. In our study, however, we looked at serum GDNF levels and then performed a cluster analysis to define PD participants' subtypes based on different GDNF levels. Next, based on the classification, we analyzed the neuroimaging data, clinical, and demographic characteristics, and cognitive features, which might elucidate brain functional changes in PD with various GDNF characteristics. Finally, we sought to create a multimodal index that may predict the early cognitive impairment (prodromal stage) of Parkinson's disease more reliably and precisely by using GDNF as the significant factor and combining it with other illness‐related characteristics and imaging data.

## 2 MATERIALS AND METHODS

### 2.1 Participants and clinical neuropsychological assessment

This case–control study was approved by the Ethics Committee of the Affiliated Hospital of Xuzhou Medical University (approval nos. XYFY2017‐KL047‐01; XYFY2020‐KL023‐01). Individuals with PD were recruited from the Neurology Department of the Affiliated Hospital of Xuzhou Medical University and assessed in‐house. Written informed consent was obtained from patients or a proxy (if necessary) for clinical data analysis and structural neuroimaging studies. Patients were included if they were fluent Chinese speakers, were aged 50–80 years, were able to give informed consent, had a diagnosis of PD according to the United Kingdom‐PD Society Brain Bank criteria, had no concomitant neurologic diseases affecting cognition (stroke, traumatic brain injury, and encephalitis), and had not undergone deep brain stimulation. Age‐ and sex‐matched controls were primarily from spouses and friends of patients: 25 controls from Xuzhou Medical University to match with cases with PD. The exclusion criteria were as follows: non‐idiopathic Parkinsonism, dementia with Lewy bodies, severe brain injury, serious illness (e.g., heart failure, psychiatric illness, and malignancy), and cognitive impairment precluding informed consent.

Thirty‐eight cases with PD and 25 controls were participants in our case–control study, in which we reported on the subjects' characteristics^20^ (See Table 1), including age, sex, formal education, physical condition and past living habits, and disease duration. The clinical variables, including the cognitive questionnaire (Mini‐Mental State Examination, Montreal Cognitive Assessment, Clinical Dementia Rating, part of the Alzheimer's Disease Assessment Scale, and the Trail Making Test‐A), Hoehn & Yahr scale, levodopa equivalent daily dose, serum GDNF level, were assessed. These variables were considered potential between‐group covariates.

**TABLE 1:** Demographic characteristics and cognition analysis of different groups.

GROUP	Healthy control (V1)	PD‐high‐GDNF (V2)	PD‐low‐GDNF (V3)	Statistic	Significance level (*p* ^a^)	*p* ^b^		
						V1:V2	V2:V3	V1:V3
Total no. of subjects	25	19	19	—	—	—	—	—
Plasm GDNF, pg/mL								
Mean, SD	517.36(140.72)	395.50(78.77)	175.86(69.12)	56.515	**0.000**	**0.002**	**0.000**	**0.000**
95%CI	459.27, 575.45	357.53, 433.47	142.54, 209.17					
Min, Max	126.05, 757.50	289.67, 575.15	111.10, 397.65					
Gender								
Male, *N* (%)	12 (48.0)	11 (57.9)	7 (36.8)	1.690	0.429	0.515	0.194	0.459
Female, *N* (%)	13 (52.0)	8 (42.1)	12 (63.2)					
Age								
Mean, SD	61.32 (5.528)	62.16 (9.714)	65.26 (4.458)	1.912	0.157	0.688	0.166	0.062
95%CI	59.04, 63.6	57.48, 66.84	63.11, 67.41					
Min, Max	53, 76	45, 79	54, 73					
Education								
Mean, SD	9 (2.872)	7.89 (3.348)	5.68 (4.083)	5.148	**0.009** *	0.292	**0.051**	**0.002** *
95%CI	7.81, 10.19	6.28, 9.51	3.72, 7.65					
Min, Max	0, 15	0, 15	0, 12					
High blood pressure *N* (%)	12 (48)	6 (31.6)	7 (36.8)	3.825	0.148	0.254	**0.051**	0.336
Diabetes *N* (%)	7 (28)	6 (31.6)	6 (31.6)	0.092	0.955	0.797	1	0.797
Smoking *N* (%)	2 (8)	5 (26.3)	5 (26.3)	3.281	0.194	0.100	1	0.100
Alcohol drinking(%)	6 (24)	3 (15.8)	9 (47.3)	5.067	0.079	0.504	**0.036** *	0.105
Disease duration, years	—	3.421 (1.169)	5.657 (3.077)	55.074	**0.000** *	**0.000** *	**0.021** *	**0.000** *
Hoehn‐Yahr (Modified) Scale								
Median (IQR)	—	1.5 (1, 2)	2 (1.5, 3)	2.121	**0.034** *	—	**0.034** *	—
0	—							
1	—	7	2					
1.5	—	3	3					
2	—	6	3					
2.5	—	1	3					
3	—	2	5					
4	—		1					
5	—							
MoCA								
Mean, SD	27.08 (1.152)	19.95 (4.552)	17.21 (3.750)	52.934	**0.000** *	**0.000** *	**0.013** *	**0.000** *
95%CI	26.6, 27.56	17.75, 22.14	15.40, 19.02					
Min, Max	25, 29	11, 27	10, 24					
MMSE								
Mean, SD	28.80 (1.00)	24.26 (3.280)	21.21 (3.441)	44.764	**0.000** *	**0.000** *	**0.001** *	**0.000** *
95%CI	28.39, 29.21	22.68, 25.84	19.55, 22.87					
Min, Max	27, 30	14, 29	13, 27					
LEDD (mg/d)								
Median (IQR)	—	200 (200, 225)	250 (200, 300)	100.90	**0.000** *	—	**0.004** *	—
Mean, SD	—	200, 70.71	263.16, 94.78					
95%CI	—	165.92, 234.08	217.47, 308.84					
Min, Max	—	0, 375	50, 500					

*Note*: Plasm GDNF, Age, Education, Disease duration, MoCA, MMSE are represented as mean ± SD unless otherwise specified. Smoking, Average number of cigarettes per day, times years. A number over 200 is considered a history of smoking. Drinking generally refers to drinking 42°C liquor, more than a bottle a week. “Bold” means *p* < 0.05 or marginal significance.

Abbreviations: MMSE, min‐mental state examination; MoCA, Montreal cognitive assessment; Pa, Between groups, Kruskal–Wallis H test; Pb, Within groups; Chi‐square, Likelihood Ratio, and Mann–Whitney U test; PD, Parkinson's disease.

* Means significant difference.

### 2.2 Plasma GDNF sample collecting and detection and cluster analysis

Patients were asked to fast from 22:00 for samples to be collected the following day. Five milliliters of blood was collected from each participant between 07:00 and 09:00. Samples were centrifuged for 10 min at 4°C at 1000*g*. Samples were kept at room temperature for up to 2 h before centrifugation. To avoid destroying the serum components, samples were immediately dispensed into 130 μL Eppendorf tubes and processed at −80°C for later assays. GDNF levels were determined using enzyme‐linked immunosorbent assay kits (GDNF ELISA Kit [human]: Cat# SEA043 Hu, Cloud‐Clone Corp) in strict compliance with the manufacturers' instructions.

The distributions of GDNF were examined using histograms and standardized for analysis using *z*‐scores. A prespecified primary cluster analysis was then performed with the GDNF values as continuous variables via the Ward minimum variance hierarchical cluster analysis method with an agglomerative approach and Ward linkage. K‐means cluster analysis was performed with the two distinctive clusters identified and also repeated with three and four clusters to assess the stability of clusters. Given the sample size, the final results identified two clusters within the PD group. We divided the dataset (PD group) into two clusters (PD‐high‐GDNF, PD‐low‐GDNF) according to the K‐means analysis.^20^ There was stability for two clusters compared with other numbers of clusters.

### 2.3 Magnetic resonance imaging acquisition

All participants were scanned using a GE3.0 Tesla (T) MRI scanner with an eight‐channel head coil (GE Medical Systems, Signa HD) at the imaging department of the Affiliated Hospital of Xuzhou Medical University. Participants were asked to remain motionless, keep their eyes closed, not think of anything, and not fall asleep during the processing. The scanning protocol includes a resting state, 3DT1 weighted structure imaging (3DT1‐WI), and blood‐oxygen‐level dependent (BOLD) imaging. (1) T1WI: repetition time/ effective echo time (TR/TE) = 6.964/2.996 ms, T1 = 2400 ms, flip angle = 12, the field of view (FOV) = 256 × 256 mm^2^, slice thickness = 1, 1 mm isotropic voxel, no interslice gap; 192 slices. (2) BOLD: TR = 2000 ms; TE = 30 ms; FOV = 220 × 220 mm^2^; slice thickness = 3 mm; slice gap = 3.5 mm, 36 slices. voxel size = 3.5 × 3.5 × 3.5, flip angle = 90, matrix = 64 × 64, 186 volumes.

### 2.4 Preprocessing of MRI data

MRI data were preprocessed using the GRETNA program and Statistical Parametric Mapping, version 12 (SPM12, http://www.fil.ion.ucl.ac.uk/spm), and the Resting‐State fMRI Data Analysis Toolkit (RESTplus) (http://www.restfmri.net) running on the MATLAB (R2013b, MathWorks) platform according to the standard procedure. Structural images were processed using voxel‐based morphometry analysis. ① Briefly, the first five time points from each subject's series were discarded due to the instability of the initial MR signals. ② This was followed by slice‐timing corrections and ③ realigning to the first volume for head motion correction. Data were excluded due to excessive head motion (>3 mm and 3°). ④ Next, the structural images were coregistered to the mean functional images after realignment, ⑤ then normalized to the Montreal Neurological Institute (MNI) brain space template (resampled voxel size 3 × 3 × 3 mm). ⑥ All images were smoothed with a 4 × 4 × 4 mm^3^ full‐width at half‐maximum (FWHM) Gaussian kernel and were linearly detrended and ⑦ bandpass‐filtered (0.01–0.08 Hz) to enable reduction of the high‐frequency respiratory and cardiac noise. ⑧ Finally, the white matter signal, cerebrospinal fluid signal, and Friston 6 head motion parameters were regressed.

### 2.5 Voxel‐wise degree centrality

We used a seed‐based approach for analysis. Multiple brain regions were selected as regions of interest (ROIs) for target seeds, including putamen, caudate, globus pallidus, medial frontal gyrus, inferior frontal gyrus, inferior temporal lobe gyrus, and inferior parietal lobule. The above ROIs mainly used target areas to focus on the difference in degree centrality.

The degree centrality, a graph theory‐based approach, and a connectivity measure of a given node in the brain network with all other nodes were calculated using DPARSF (http://rfmri.org/dparsf). The individual Pearson's correlation coefficients were computed in a prior probability brain gray matter mask in SPM8 between the time course of a given voxel and all other whole‐brain voxels within the template. We studied each voxel of Pearson's correlation coefficients after extracting the BOLD time course (defined as correlation coefficient *r* > 0.25) in the entire brain. Then the binary DC values of the whole‐brain network were obtained. These maps were then z‐transformed to enable group comparisons after all individual DC maps were spatially smoothed with a Gaussian smoothing kernel. DPABI 4.0 software (Matlab2013b) was used to calculate the DC value of the brain region of the differential clusters in this project.

### 2.6 Cortical thickness and graph theory analysis of structural covariation networks

According to the aparc template, the cortex was divided into 68 brain regions, and then the cortex thickness was calculated using freesurfer (http://surfer.nmr.mgh.harvard.edu/fswiki/CorticalParcellation), which has been used widely to perform an automatic volumetric calculation. Freesurfer is an automated morphology program that identifies brain white matter and gray matter, generating a white/gray matter boundary. The cortical thickness of the cortical surface area of interest was calculated using the Euclidean distance between the linked vertices of the inner and outer surfaces.

The two fundamental elements of the network are edges and nodes, where nodes represent the brain regions and edges depict the functional connectivity between two brain regions or nodes. Using the GRETNA toolbox, a 68 × 68 covariant functional network matrix was constructed to compute a structural correlation network for three groups. Next, each network was binarized over sparsity ranges from 10% to 40% at 0.01 intervals. A maximum of 40% and a minimum of 10% sparsity were determined based on the small‐world property (sigma), ensuring that the sigma values for each group of all our subjects were just above 1.1 (sigma > 1.1).^22^ When the sparsity is <10%, the sigma value will drop sharply, and the sigma value cannot be guaranteed to be greater than 1.1. Based on this, we used 10%–40% sparsity for other network attributes. The sparsity ranging from 10% to 40% improved the effect of neural network topologies in our study. Finally, we compared the network topologies based on the theory analysis. The global topological metrics of the network analysis, including the clustering coefficient (Cp), characteristic pathlength (Lp), small‐worldness (a metric reflecting the degree of network economic optimization and traditionally characterized by Cp and Lp, Sigma), and global efficiency (Eglob), and local efficiency (Eloc), were calculated. Local network graph metrics were calculated and compared among three groups, including betweenness, degree centrality, and local efficiency. The area under the curve (AUC) for each network metric providing a summarized scalar for topological properties was calculated in this study. A regression analysis corrected the effects of age, sex, and education before nonparametric the 1000 permutation tests were undertaken.

### 2.7 Statistical analysis

The demographic and clinical data were compared using SPSS 22.0 software, and the Kolmogorov–Smirnov test was applied to assess data normality. Two‐tailed independent‐sample *t*‐tests and analysis of variance (ANOVA) were used for the normally distributed variables. Non‐normally distributed data were evaluated using the Kruskal–Wallis H test. The Chi‐squared test was used to compare the sex distribution between groups. We performed the correction of multiple comparisons using the Bonferroni correction method. The significance level was set as *p* < 0.05.

We combined DC and target regions of interest to evaluate specific regional differences in DC among the HC, PD‐high‐GDNF, and PD‐low‐GDNF groups. The DC values of voxels in the ROI clusters were extracted using SPM8. First, the Kolmogorov–Smirnov test was used to evaluate the typical distribution characteristics of the DC values of clusters. ANOVA and post hoc two‐sample t‐tests in a pairwise manner within the areas identified by the ANOVA were used to identify the differential brain regions among the three groups. Pearson's correlation analysis estimated the associations between DC values of different brain cluster regions and neuropsychological data. The area under the receiver operating characteristic curve (AUROC) and the Youden‐Index (Sensitivity + specificity) were used to report the threshold probabilities in the predictive values of the cognitive dysfunction status. The significance level was set at *p* < 0.05 (two‐tailed). Cross‐validation is, in this case with small datasets, a typical strategy for estimating the performance. To validate our results, we performed a cross‐validation analysis in R software. In the 10‐fold cross‐validation analysis, the sample was randomly divided into 10 batches. In each run, one batch was used as the testing data and the remaining nine as training data. A generalized linear model was applied to each test dataset to obtain prediction accuracy. The process was repeated 10 times, with each of the 10 samples used once as the testing data. Then the testing dataset with the maximum accuracy was used to construct the final generalized linear model, and the remaining training dataset was used to calculate the prediction values of the model and draw the ROC curve.

A nonparametric permutation test with 1000 permutations was used to assess the difference between global graph metrics and local network metrics. In addition, linear regression models corrected the graph metrics and the cortical thickness values for age and sex effects. Pearson's correlation coefficient was used to correlate significantly different regional cortical and total cognitive function scores, including the subdomain of cognition, which was used to investigate the key cortical regions in the cognitive function of PD. The ultimate target was the core brain region with the most associated cognitive domain scores. Finally, AUROC analyses were performed to investigate the targeted cortical thickness values in our cohort's mental level.

Graphical and statistical analyses were performed using MATLAB (version R2013b) and SPSS version 22.0 (SPSS Inc., Chicago, IL, USA).

### 2.8 Study approval

The clinical study was performed following principles of the Declaration of Helsinki. All clinical samples collected were approved by the Ethics Committee of the Affiliated Hospital of Xuzhou Medical University. All patients provided written informed consent before enrollment in the study.

## 3 RESULTS

### 3.1 Altered global properties of brain network

**FIGURE 1:**
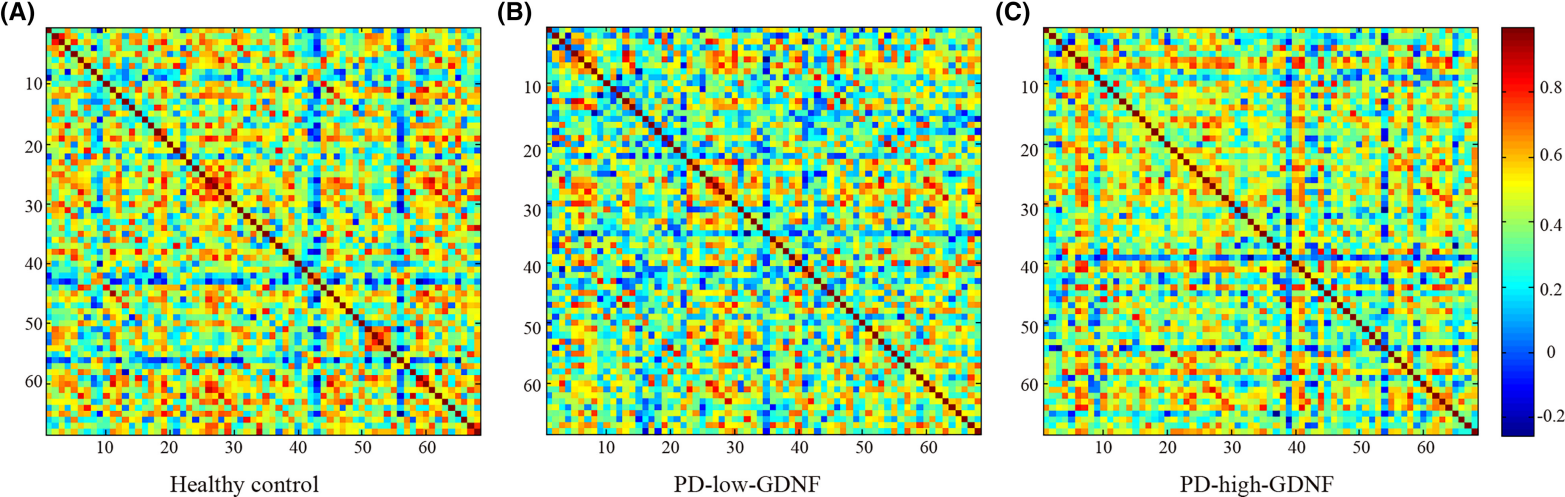
Brain inter‐regional correlation matrices for HC (A) and PD (B: PD‐low‐GDNF, C: PD‐high‐GDNF) groups. The matrix (68 × 68) shows the Pearson correlation coefficient between any two nodes of the network. The color bar represents the absolute value of the Pearson correlation coefficient, which ranges from 0 (blue) to 1 (red).

**FIGURE 2:**
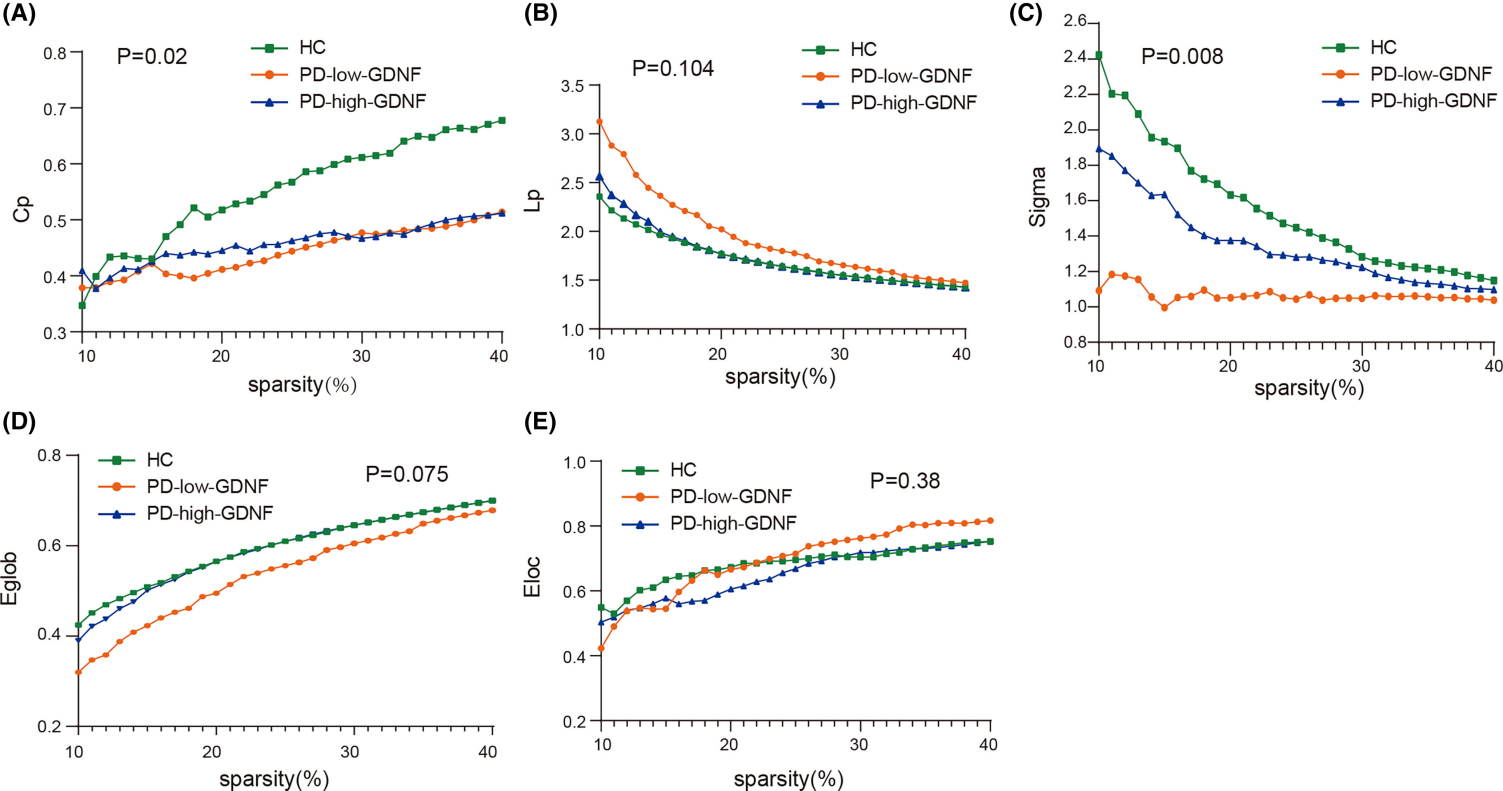
Changes in the global network parameters of the structural covariance network as a function of network sparsity. (A) Clustering coefficient, Cp; (B) characteristic Pathlength, Lp; (C) small‐world index, sigma; (D) global efficiency, Eglob; (E) local efficiency, (Eloc)in healthy controls (HCs), PD‐high‐GDNF, and PD‐low‐GDNF patients.

Based on the clustering analysis, we separated the participants into healthy controls, PD‐high‐GDNF, and PD‐low‐GDNF. All three groups were well‐matched. All participants' demographic and clinical characteristics are shown in Table 1 (The data in Table 1 have also been published in the journal *Neural Regeneration Research*. We used the same participants for the imaging study). As theoretical graph approaches have developed, alternative metrics based on brain network topology have received growing attention. Group differences were explored using the nonparametric permutation test.^23^ In the current covariant structural connection analysis, the correlation matrix of the three groups was obtained by constructing the 68 × 68 Pearson correlation coefficient of the cerebral cortex (Figure 1). The correlation matrix of each group was converted into a binary network matrix with a fixed sparsity threshold of 10%–40%. The graph structure network was formed, and the topology properties of the network were analyzed. Over the sparsity range 0.10–0.40 (step =0.01), both the PD‐high‐GDNF and PD‐low‐GDNF groups exhibited reduced Cp (*p* = 0.019) and low‐efficiency small‐world topology (sigma = gamma (Cp)/ lambda (Lp); *p* = 0.008) (Figure 2; Table 2). The Eglob value of the PD‐low‐GDNF group was decreased compared to the HC group, although it was statistically marginal significant (*p* = 0.06). The Eloc value of the three groups was no different (*p* = 0.380). However, PD‐low‐GDNF showed increases in the characteristic path length and Eloc at the partial sparsity range in the network's modularity. Notably, the PD‐low‐GDNF group showed low global efficiencies, which indicates less efficient information transmission over a global network. It also implies that subjects in the PD‐low‐GDNF group show a poor ability to separate and integrate information.

**TABLE 2:** Statistical comparisons of the global graph metrics of the structural covariance network.

Global metrics of the structural networks	AUC			*p* value	HC vs PD‐low‐GDNF	HC vs pd‐high‐GDNF	PD‐low‐GDNF vs PD‐high‐GDNF
	HC	PD‐low‐GDNF	PD‐high‐GDNF				
Cp, clustering coefficient	0.1607	0.1331	0.1374	**0.019**	**0.009**	**0.014**	0.861
Lp, shortest path length	0.5124	0.5793	0.5193	0.104	**0.038**	0.957	**0.079**
Eglob, global efficiency	0.1787	0.1617	0.1772	**0.075**	**0.06**	0.096	0.849
Eloc, local efficiency	0.2045	0.1957	0.2090	0.380	0.163	0.595	0.404
Gamma	0.4765	0.3287	0.4179	**0.047**	**0.019**	0.145	0.324
Lambda	0.3079	0.3095	0.3082	0.970	0.838	0.957	0.79
Sigma, small world	0.4618	0.3199	0.4028	**0.008**	**<0.001**	**0.01**	**0.008**

*Note:* “Bold” means *p* < 0.05 or marginal significance.

### 3.2 There are disparities in the degree of centrality in the resting state between groups, and the correlation with cognitive performance is evident

**FIGURE 3:**
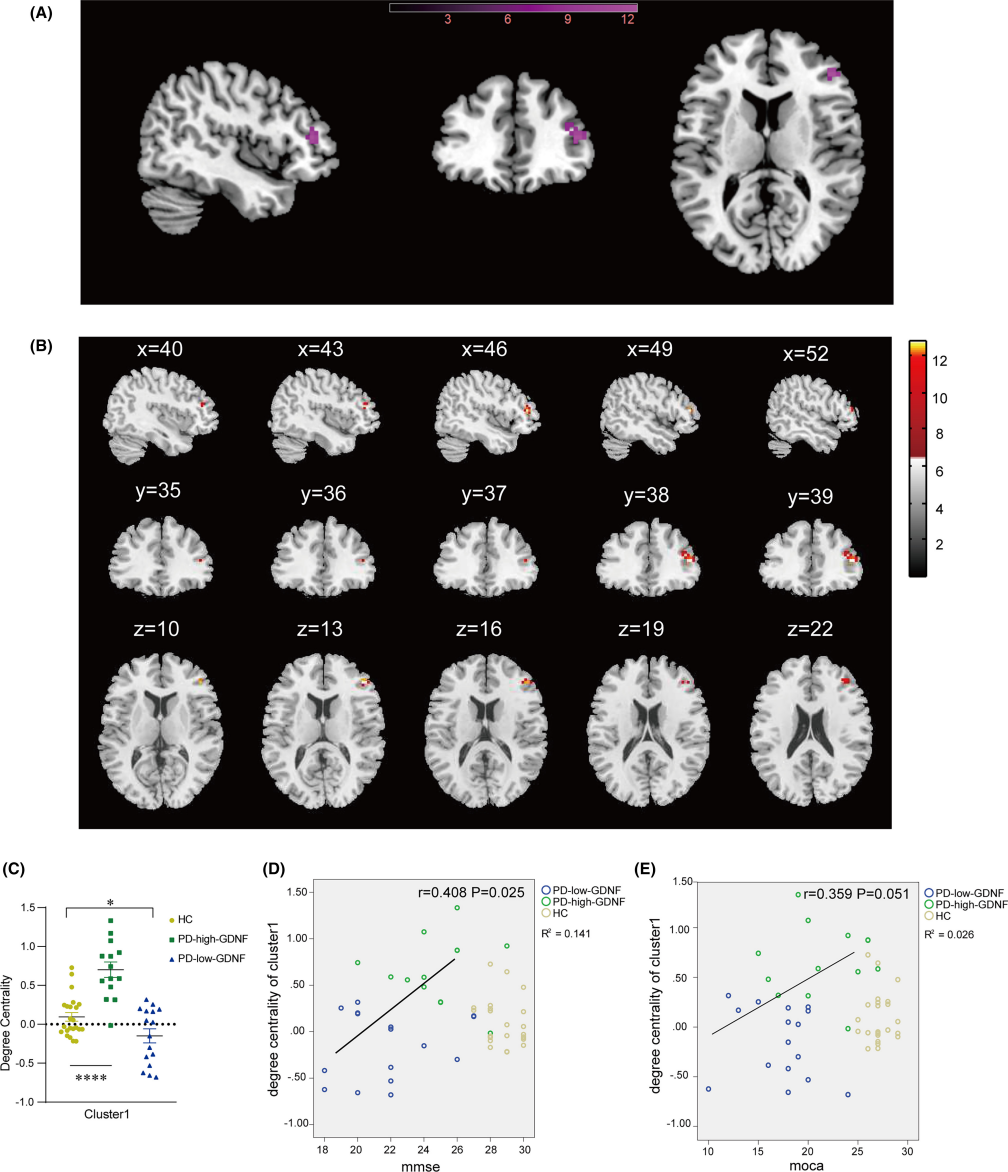
Brain areas showing the difference in the degree centrality of cluster 1 among three groups and their correlations with clinical cognition features in all subjects. (A) Rendering views of cluster 1 (B) axial slice views. Color bars indicate *F*‐value. The coordinate region is mainly in the frontal lobe of the right cerebrum. The DC cluster was performed at the threshold of *p* < 0.05, and corrected for multiple comparisons via the false discovery rate (FDR) method. (C) The degree of centrality values of among‐group differences in cluster1. *p < 0.05; ****p < 0.0001. (D, E) The DC of the cluster 1 scatter plot in all subjects and correlations with MMSE and MoCA scores in all PD subjects.

**FIGURE 4:**
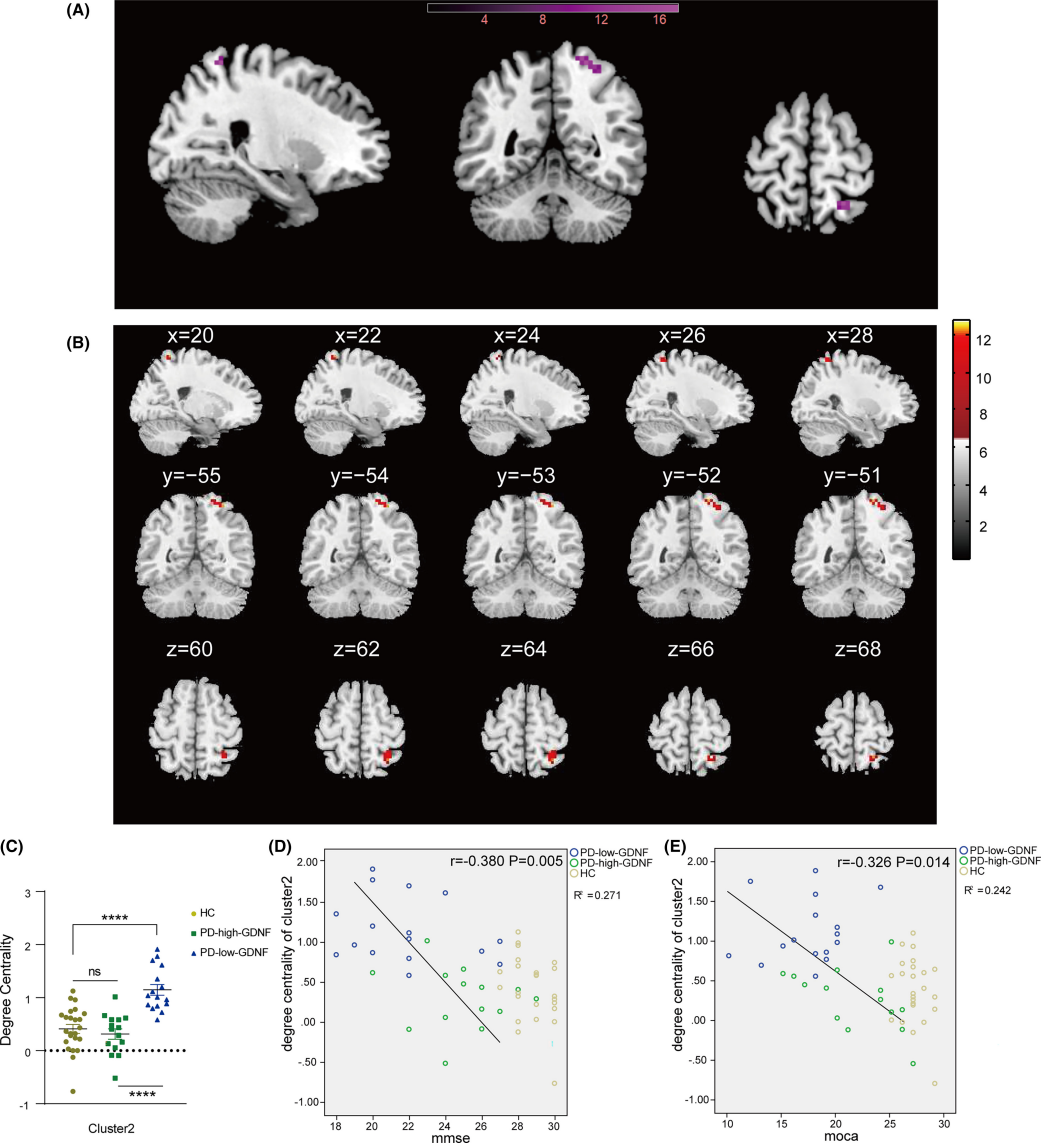
Brain areas showing the difference in the degree centrality of cluster 2 among three groups and their correlations with clinical cognition features in all subjects. (A) Rendering views of cluster 2 (B) axial slice views. Color bars indicate *F*‐value. The coordinate region is mainly in the parietal lobe of the right cerebrum. The DC cluster was performed at the threshold of *p* < 0.05, and corrected for multiple comparisons via the false discovery rate (FDR) method. (C) The degree of centrality values of among‐groups differences in cluster1. *p < 0.05; ****p < 0.0001, ns, non‐significant difference. (D, E) The DC of the cluster 2 scatter plot in all subjects and significant correlations between DC and cognitive function (MMSE, MoCA) in PD subjects.

The voxel‐based degree centrality analysis on pre‐defined key brain regions shows that two clusters of abnormalities were identified on the brain map after FDR correction (Table 3). Cluster1 of voxels within the right cerebrum Frontal lobe Inferior Frontal Gyrus was altered, with a little extension to the gyrus frontalis mediums (Brodmann area 46, 10, BA) (Figure 3A,B). Compared with HC, PD individuals showed significantly enhanced DC in cluster 1. Notably, PD with high serum GDNF showed increased DC for cluster 1. In contrast, PD with low GDNF showed a remarkable decrease (Figure 3C). Subsequently, the correlation analysis between the PD individual DC of cluster1 and the MMSE (*r* = 0.408, *p* = 0.025), and MoCA (*r* = 0.359, *p* = 0.051) scores revealed a positive correlation (Figure 3D,E). Cluster 2 was observed in the regions mainly in the Right Cerebrum Parietal Lobe Postcentral Gyrus (Brodmann area 7, 5) (Figure 4A,B). Compared to HC, the PD‐high‐GDNF showed no change. In contrast, PD‐low‐GDNF showed an increased DC in cluster2 (Figure 4C). Interestingly, scatter plots of the correlation between the DC of cluster2 and the cognition scores, other than cluster1, indicated a marked negative correlation with MMSE (*r* = −0.380, *p* = 0.005) and MoCA (*r* = −0.326, *p* = 0.014) (Figure 4D,E).

**TABLE 3:** Regions showing significant differences in degree centrality among three groups.

Items	Brain regions	MNI	Location	Peak intensity	Voxels
Cluster 1	Right Cerebrum Frontal Lobe Inferior Frontal Gyrus	45, 39, 12	BA 46, 10	12.3997	20
Cluster 2	Right Cerebrum Parietal Lobe Postcentral Gyrus	24, −51, 66	BA 5, 7	17.1576	27

*Note*: Significant thresholds were corrected using FDR criterion and set at *p* < 0.01.

Abbreviations: BA, Brodmann's area; MNI, Montreal Neurological Institute.

### 3.3 Analysis of ROC of degree centrality values

**FIGURE 5:**
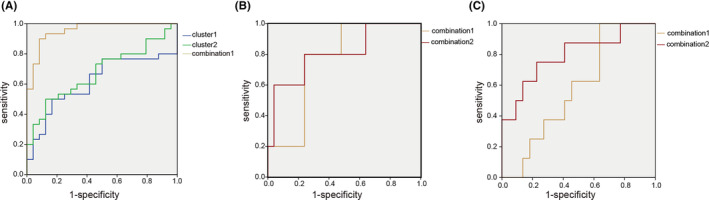
Receiver operating characteristic (ROC) curves of degree centrality single indicator and composite index obtained when predicting Parkinson's disease and varying degrees of cognitive impairment based on the judgment of MMSE and MoCA. (A) Validation of ROC curves for Parkinson's disease diagnosis. (B) In the PD subgroup, validation ROC curves for moderate cognition impairment are based on the MMSE score. (C) In the PD subgroup, validation ROC curves for mild cognitive impairment are based on the MoCA score. Cluster 1: blue line; cluster 2: green line; Combination 1 (brown line): DC of clusters 1 and 2, serum GDNF; combination 2 (red line): duration, LEDD, serum GDNF, and DC of clusters 1 and 2.

Following the remarkable findings of the correlation analysis, we hypothesized that the DC discrepancies between clusters 1 and 2 could be a possible diagnostic indicator in detecting PD and different cognitive statuses. As a result, the receiver operating characteristic (ROC) curve approach was used to assess the DC values in clusters 1 and 2. In the ROC analysis, the areas under the ROC curve (AUC) for clusters 1 and 2 were 0.615 (95% CI: 0.461, 0.769, *p* = 0.149), and 0.676 (95% CI: 0.533, 0.819, *p* = 0.027), respectively. Next, we analyzed the correlation between GDNF, DC value, and cognitive assessment score (Figure S1). In the PD group, Cluster1 DC was positively correlated with serum GDNF levels, and DC was positively correlated with cognitive score. In other words, the higher the serum GDNF in PD, the higher connectivity of cluster1 and the better the cognition status. Similarly, serum GDNF level is negatively correlated with cluster2 DC, and DC is negatively correlated with cognition in PD population (Table S1). In other words, the higher the serum GDNF of PD patients, the lower the cluster2 connectivity and the better the cognitive evaluation. Given the significant correlation characteristics of the serum GDNF and DC values within the PD group, we speculate that combined detection can improve the accuracy of screening for the cognitive impairment of PD patients. Indeed, the AUC value was 0.956(95%CI: 0.906, 1.00, *p* = 0.000) after cluster1, cluster2, and serum GDNF markers were combined (Figure 5A; Table 4), a significant improvement over the AUC results obtained for a single indicator. We conducted a cross‐validation analysis and found the results were consistently significant in the validation tests, indicating the robustness of our findings.

**TABLE 4:** The results of ROC analysis for judging Parkinson disease.

Variable	AUC (95% CI)	Standard error	Optimal cut‐off	Sensitivity (%)	Specificity (%)	Youden's index (%)	Asymptotic significance
Cluster 1	0.615 (0.461, 0.769)	0.079	0.2983	50.0	83.3	33.3	0.149
Cluster 2	0.676 (0.533, 0.819)	0.073	0.7857	50.0	87.5	37.5	**0.027**
Combination 1	0.956 (0.906, 1.000)	0.025	0.5358	93.3	87.5	80.8	**0.000**

*Note*: “Bold” means *p* < 0.05 or marginal significance.

Further ROC analysis assessed the DC values as the severity of cognitive impairment diagnostic tool for Parkinson's disease. Based on MMSE scores, the PD group was classified into mild and moderate cognition impairment groups. When these two groups were compared, the AUC of combination indicator 2 (DC of clusters 1 and 2, serum GDNF, duration, LEDD) was 0.808 (95% CI: 0.581, 1.00, *p* = 0.032, Youden's index = 56%), but the independent indicators, that is, 3‐biomarker combination 1 (DC of cluster 1 and 2, serum GDNF), could not identify the cognitive status (Figure 5B,C; Table 5). Similarly, 3 biomarker combination failed to identify the mental level after the Moca assessment. The optimal diagnostic biomarker combination‐ 2(DC of clusters 1 and 2, serum GDNF, duration, LEDD) proved to be the most accurate combination assay for the detection of cognitive status (AUC = 0.795, 95%CI: 0.601, 0.990, *p* = 0.015, Youden's index = 52.3%) (Table 6). Similarly, we cross‐validated the ROC of the final combined variables, and the model was reliable.

**TABLE 5:** The results of ROC analysis for cognition status judged by MMSE in PD.

Variable	AUC (95% CI)	Standard error	Optimal cut‐off	Sensitivity (%)	Specificity (%)	Youden's index (%)	Asymptotic significance
Combination 1	0.760 (0.571, 0.949)	0.096	0.2202	80.0	76.0	56.0	0.071
Combination 2	0.808 (0.581, 1.000)	0.116	0.1705	80.0	76.0	56.0	**0.032**

*Note*: Combination 1: DC of clusters 1 and 2, serum GDNF. Combination 2: Duration, LEDD, serum GDNF, and DC of clusters 1 and 2. “Bold” means *p* < 0.05 or marginal significance.

**TABLE 6:** The results of ROC analysis for cognition status judged by MoCA in PD.

Variable	AUC (95% CI)	Standard error	Optimal cut‐off	Sensitivity (%)	Specificity (%)	Youden's index (%)	Asymptotic significance
Combination 1	0.580 (0.372, 0.788)	0.106	0.2082	100.0	36.4	36.4	0.511
Combination 2	0.795 (0.601, 0.990)	0.099	0.3398	75.0	77.3	52.3	**0.015**

*Note*: Combination 1: DC of clusters 1 and 2, serum GDNF. Combination 2: Duration, LEDD, serum GDNF, and DC of clusters 1 and 2. “Bold” means *p* < 0.05 or marginal significance.

### 3.4 Cortical thickness differences were mainly concentrated in the frontal and temporal lobes and correlated with the clinical cognition assessment

**FIGURE 6:**
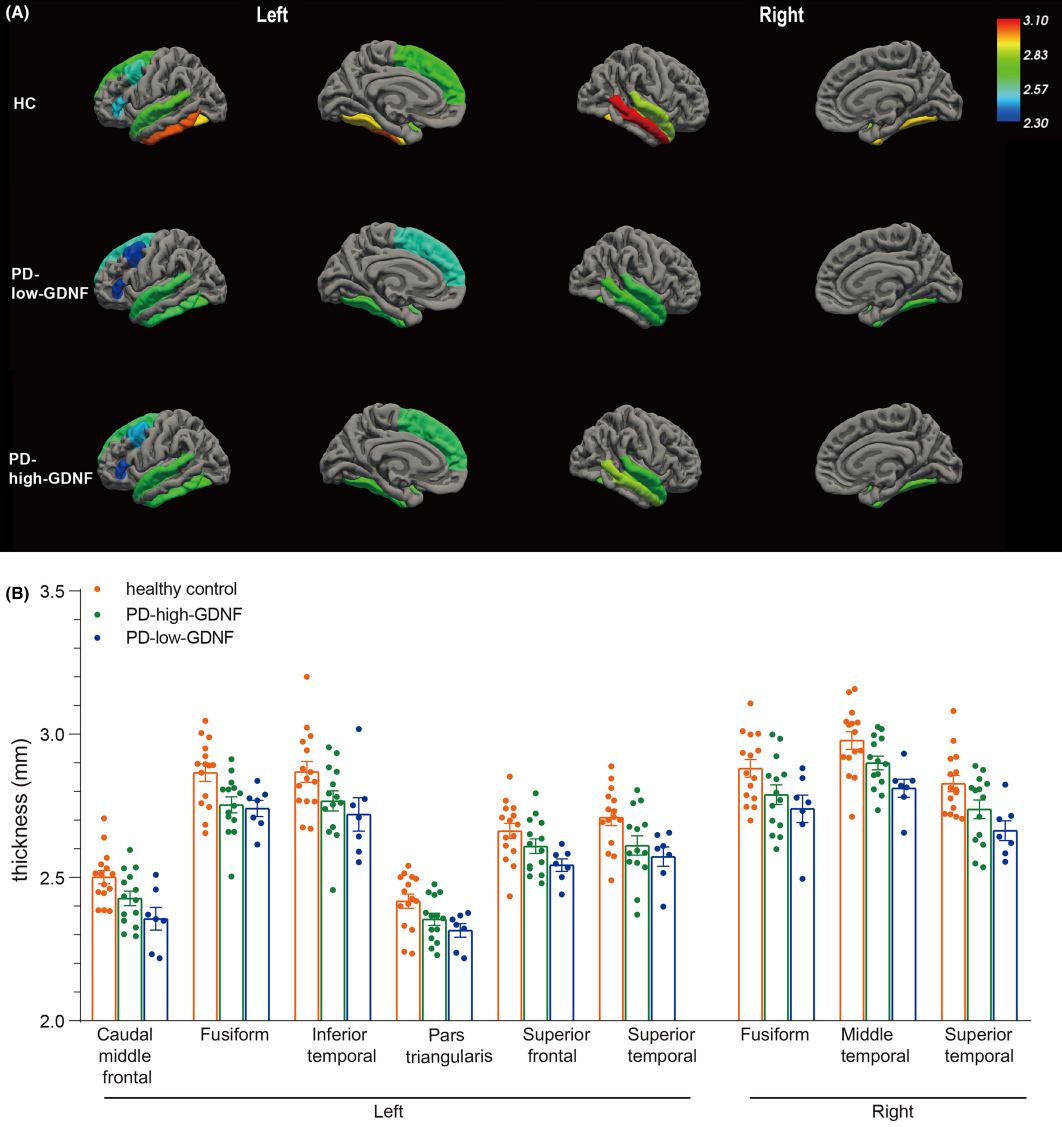
Group cortical thickness differences. (A) Cortical structures, where statistically significant changes are marked in different colors to distinguish each cortical region's thickness. Results from the analysis of cortical thickness showing cortical thinning in PD patients (PD‐high‐GDNF and PD‐low‐GDNF) compared with healthy controls are displayed in significantly different regions of a standardized brain (averaged over all subjects). In particular, the cortical thickness of the nine brain areas is decreased considerably in the PD‐low‐GDNF group. (B) The statistical comparisons were analyzed, and the nine regions listed were all statistically different based on the one‐way ANOVA method, *p* < 0.05.

Cortical thickness was measured to reflect the cortical structural morphometry directly. The patterns of brain regions with differences in cortical thickness between three groups are shown in Figure 6A. Mean values of cortical thickness appear to be decreased in the left Caudal middle frontal cortex, left Fusiform, left inferior temporal, left Pars triangularis, left superior frontal, left superior temporal, right Fusiform, right middle temporal, and right Superior temporal regions in the PD group compared to HC group. More specifically, the PD‐low‐GDNF group has a reduced thickness among these three groups (Figure 6B; Table 7).

**TABLE 7:** Group cortical thickness differences and comparison.

		Thickness (mean ± SD)			*F* statistic	*p* value	Multiple comparisons‐LSD		
		HC	PD‐high‐GDNF	PD‐low‐GDNF			HC vs PD‐high‐GDNF	HC vs PD‐low‐GDNF	PD‐low‐GDNF vs PD‐high‐GDNF
Left	Caudal middle frontal	2.50 ± 0.09	2.42 ± 0.09	2.35 ± 0.11	5.855	0.007	0.045	0.002	0.117
	Pars triangularis	2.42 ± 0.09	2.35 ± 0.07	2.3 ± 0.063	4.001	0.028	0.053	0.013	0.332
	Superior frontal	2.66 ± 0.10	2.60 ± 0.09	2.54 ± 0.05	3.978	0.028	0.133	0.009	0.140
	Fusiform	2.86 ± 0.11	2.75 ± 0.10	2.74 ± 0.07	5.375	0.010	0.007	0.014	0.795
	Inferior temporal	2.86 ± 0.14	2.76 ± 0.13	2.71 ± 0.15	3.347	0.047	0.059	0.027	0.475
	Superior temporal	2.70 ± 0.10	2.61 ± 0.12	2.57 ± 0.08	4.550	0.018	0.025	0.012	0.458
Right	Fusiform	2.88 ± 0.12	2.80 ± 0.14	2.75 ± 0.11	3.664	0.037	0.055	0.019	0.376
	Middle temporal	2.98 ± 0.12	2.89 ± 0.09	2.81 ± 0.08	6.620	0.004	0.046	0.001	0.072
	Superior temporal	2.83 ± 0.11	2.73 ± 0.12	2.66 ± 0.09	5.699	0.007	0.037	0.003	0.156

We then undertook a correlation analysis between cortical thickness and clinical cognition scores. The Person correlation results revealed that not all the different brain regions were significantly correlated with cognitive scores from MMSE or MoCA. Still, only six cortical areas were positively correlated with the cognitive results (Table 8).

**TABLE 8:** Person Correlation between cognitive function score and cortical thickness.

		L_caudal middle frontal	L_fusiform	L_inferior temporal	L_pars triangularis	L_superior frontal	L_superior temporal	R_fusiform	R_middle temporal	R_superior temporal
mmse	*r*	0.250	0.191	0.129	0.248	0.166	0.240	0.260*	0.164	0.174
	*p*	**0.040**	0.116	0.289	**0.042**	0.173	**0.049**	**0.033**	0.177	0.152
moca	*r*	0.278	0.160	0.086	0.228	0.239	0.150	0.219	0.251	0.058
	*p*	**0.022**	0.187	0.475	0.060	**0.048**	0.216	0.070	**0.038**	0.630

*Note*: L, Left hemisphere; R, Right hemisphere; *r*, Pearson's correlation coefficient; “Bold” means *p* < 0.05.

Spearman correlation analysis was performed to clarify which cortex region was most closely relevant to a subdomain of cognition. The results showed that the thickness of the left caudal middle frontal was positively correlated with orientation, memory, attention, and executive function (Table 9). Further, there was a marginal statistical difference in the Correlation with the naming function (*p* = 0.051). In addition, the superior frontal, superior temporal, and fusiform regions demonstrated significant correlations between cortical thickness and a small part of the sub‐domain scores (Table 9). Within the six regions, the left caudal middle frontal region was associated with multiple cognitive domains, which indicated that patients with a thicker cortex in the left caudal middle frontal region performed better in cognition behavior, especially in respect of executive function.

**TABLE 9:** Spearman correlation analysis between cognition sub‐domain scores and different cortical thickness.

		L_caudal middle frontal		L_superior temporal		L_superior frontal		L_pars triangularis		R_fusiform		R_middle temporal	
		*r*	*p*	*r*	*p*	*r*	*p*	*r*	*p*	*r*	*p*	*r*	*p*
Orientation	MMSE (10)	0.361	**0.015**	0.111	0.259	0.346	**0.019**	0.143	0.203	0.105	0.272	0.364	**0.015**
	MoCA (6)	0.440	**0.004**	0.391	**0.009**	0.370	**0.013**	0.336	**0.023**	0.339	0.022	0.250	0.071
Memory	3 Word Immediate memory of MMSE (3)	0.441	**0.004**	0.312	**0.032**	0.351	**0.018**	0.257	0.065	0.164	0.170	0.228	0.090
Attention	Backwards digit‐span (5)	0.369	**0.013**	0.200	0.122	0.195	0.127	0.228	0.091	0.266	**0.059**	0.116	0.250
	Tap testing of MoCA (1)	0.187	0.137	0.136	0.214	0.226	0.093	−0.017	0.461	−0.013	0.471	0.060	0.365
	Digit backward of MoCA (2)	0.025	0.442	−0.090	0.300	0.016	0.463	0.017	0.460	−0.002	0.496	−0.108	0.264
Language	Boston Naming Test (30)	0.277	**0.051**	0.275	**0.052**	0.125	0.234	0.207	0.113	−0.002	0.104	0.205	0.115
	Repeat of MoCA (2)	0.249	0.072	−0.041	0.407	0.188	0.136	0.067	0.349	−0.125	0.234	0.160	0.176
Executive	Clock Drawing Test (4)	0.284	**0.046**	0.158	0.179	0.260	**0.063**	0.252	0.069	0.285	**0.046**	0.108	0.264
	TMT‐A	−0.087	0.306	0.056	0.374	−0.221	0.097	−0.123	0.237	−0.109	0.263	−0.068	0.346

*Note*: L, Left hemisphere; R, Right hemisphere; *r*, Spearman correlation coefficient; “Bold” means *p* < 0.05 or marginal significance.

### 3.5 ROC analysis of left caudal middle frontal thickness for altered cognition levels

**FIGURE 7:**
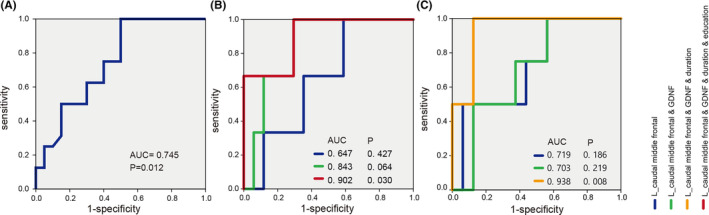
Receiver operating characteristic (ROC) curves of L_ caudal middle frontal thickness single indicator and composite index obtained when predicting Parkinson's disease and varying degrees of cognitive impairment based on the judgment of MMSE and MoCA. (A) Validation of ROC curves for Parkinson's disease diagnosis according to L_ caudal middle frontal thickness. (B) In the PD subgroup, validation ROC curves for moderate cognition impairment are based on the MMSE score. (C) In the PD subgroup, validation ROC curves for moderate cognitive impairment are based on the MoCA score. L_ Caudal middle frontal thickness: blue line; L_ caudal middle frontal thickness and serum GDNF: green line; L_ caudal middle frontal thickness, serum GDNF, and duration: yellow line; L_ caudal middle frontal thickness, serum GDNF, duration, and education: red line.

Considering the importance of the left middle frontal gyrus and its significant correlations with cognition, we used ROC analysis to evaluate whether the biomarker could be more sensitive in predicting the disease or the severity of cognition impairment in Parkinson's. The results showed that the AUC was 0.745 (95% CI: 0.586, 0.905) (Figure 7A), with a specific value of 50% and a sensitivity of 100% in discriminating PD patients from non‐PD patients. It did not, however, predict the degree of cognition impairment using only the left caudal middle frontal thickness. Based on the MMSE assessment criteria for cognition, the AUC increased from 0.647 (left caudal middle frontal thickness only) to 0.843 (left caudal middle frontal thickness variable plus GDNF) and 0.902 (*p* = 0.030, left caudal middle frontal thickness variable plus GDNF, duration, and education) (Figure 7B). Sensitivity and specificity were both 100% and 70.6%, respectively. Using the MoCA assessment criteria for cognition, we incorporated left caudal middle frontal thickness and serum GDNF duration into a multivariable prediction model, and the AUC rose from 0.719 to 0.938 (*p* = 0.008) (Figure 7C). Sensitivity and specificity increased to 100% and 87.5%, respectively. Likewise, the ROC model of the final combined variables was cross‐validated, and we found that the results were consistently significant in the validation tests, indicating the robustness of our models.

## 4 DISCUSSION

To the best of our knowledge, this is the first study to use fMRI scans to investigate the brain network topological properties and cortical thickness in Parkinson's disease patients with varying GDNF levels, and to discover that the observed aberrations in globe/regional network properties, degree centrality, and thickness difference are related to serum GDNF and cognition status.

Algebraic topology has been applied to elucidate the function of neural systems.^11^ In our structural covariant network analysis based on voxels, we observed that the global statistics of the network show a change in small‐world parameters (Cp, Lp, γ, σ). In the PD‐low‐GDNF group, the results show brain networks with reduced levels of small‐worldness. Cp was significantly lower than in the HC group, and Lp was increased, which signifies insufficient information /integration processing because of the decline in combining technical information from different brain regions or functional integration.^24^ The Eglob was also used to reflect the efficiency of data exchange.^21^ Eg was significantly lower than in the HC group due to its susceptibility to longer Lp, which suggests more difficult information integration across the brain. Nodal metrics showed abnormal connection alterations in the inferior parietal lobule and transverse temporal gyrus, which suggests the two regions could be regarded as sensitive observation areas for nodal topological attributes in PD patients with high/low GDNF levels. In relation to the cognitive performance of the patients included in this study and their relationship between GDNF and cognition, it can be concluded that the alterations in brain topological attributes, especially in connectivity attributes, in PD with low serum GDNF may be related to the occurrence of cognitive decline.

The neural function operates at multiple scales, from individual cell connections to local anatomical areas and larger brain regions connected through neural pathways. Network connectome analysis allows us to measure cognitive processes in the brain. By examining changes in local properties, we can analyze the degree centrality of a specific region of interest. The whole‐brain network centrality analysis revealed significantly different DC in our groups' BA 46, 10 (cluster1) and BA 7, 5 (cluster2) regions. The post hoc pairwise study showed that cluster 1 exhibited significantly lower DC values in the PD‐low‐GDNF patients than in the HC group, while there were higher DC values in the PD‐high‐GDNF group than in the HC group. Interestingly, the cluster1 DC values of the participants and cognition scores presented an inverted U‐shaped relationship. BA46 and BA10, part of the frontal cortex, roughly correspond with the dorsolateral prefrontal cortex (DLPFC). Studies have shown that lesions in the DLPFC contribute to delayed–responses in tasks with spatial auditory cues.^25^

Additionally, some studies have demonstrated the critical role of the DLPFC in verbal and nonverbal semantic cognition.^26^ After reviewing the cognitive status, we found that PD‐high‐GDNF subjects tended to have mild cognitive impairment. In contrast, patients with PD‐low‐GDNF tended to have moderate cognitive impairment. Therefore, we speculated that the DC value of cluster1 represented a slight increase in initial MCI patients and a severe decline with the aggravation of cognitive impairment. The increased DC seen in the DLPFC of MCI may represent a compensatory mechanism that enables patients to perform generally according to their cognitive subdomain testing. Gigi et al. also confirmed the over‐activity in the DLPFC of MCI with average semantic memory performance.^27^ Comparing cognitive subdomains between PD‐high‐GDNF and PD‐low‐GDNF, disturbances in language and executive function modulated by the DLPFC in the semantic network on working memory were found.^26^ In one quantitative electroencephalography of PD study, it was also found that the electrical activity of the frontal lobe was abnormal,^28^ which suggests cortical synaptic injury or loss in PFC. Through our analysis, we have more confidence in believing the role of the DC value of cluster1 in the modulation of the whole cognition level, specifically language and executive function. The findings in this study support the contention that frontal lobe damage is a common pathology in PD with cognitive dysfunction.^29^

Cluster 2 was located in the right cerebrum parietal lobe (PL) postcentral gyrus (Brodmann's area 7, 5). BA 7 is involved in finding objects in space and serves as a point of convergence between vision and proprioception to determine where things are in relation to parts of the body.^30^ BA5, a subdivision of the parietal lobe, is implicated in humans' sensorimotor control of hand movement.^31^ Under BA7 and BA5's extensive connectivity, these regions participate in multiple cognitive processes, such as spatial attention and navigation, decision‐making, and working memory.^32^, ^33^ In our study, the DC value of cluster2 in PD patients was increased, specifically in PD‐low‐GDNF subjects. The value was negatively correlated with the assessment of cognitive function. It represented an ‘excessive connection’ in the parietal lobule in PD‐low‐GDNF patients with worse cognitive performance. Given the cluster 1 decrease in PD‐low‐GDNF patients, we speculated that the functional connectivity of the frontal lobe was significantly decompensated (the PD‐high‐GDNF group could be fully compensated), which was probably caused by the loss of structural synaptic connections.

In addition, with the decrease in the global network connectivity, the ectopic local module interconnection increases according to higher Eloc. Therefore, we hypothesized that increased connectivity (cluster2) might be a pathological response to the long‐range connectivity deficit due to short‐range regional module compensation.^34^ In other words, this phenomenon occurs when patients with poor executive function need to increase their abnormal regional connection to contend with cognition deficits. Critically, one study regarding attention deficits reported increased centrality of the PL in attention‐deficit/hyperactivity disorder patients.^35^ In a PD patients walking research, it also showed that increased effective connectivity of the parietal lobe of PD patients played a compensatory role.^36^ Yet, whether the IPL exists as an abnormal functional connection in PD‐low‐GDNF subjects remains unknown. One possibility is that an improperly connected IPL hub disrupts neural systems during task performance in real life, thereby causing impairments in executive functioning, including attention and working memory. These results suggested that the alterations of PFC and PL might be involved in the pathogenesis in patients with cognitive dysfunction.

Serum GDNF has been regarded as the main factor in this study. We combined DC values and other variables and confirmed the practical utility of the MMSE/ MoCA‐based cognition classifier. The three factors will accurately predict whether or not a patient has PD. Surprisingly, we further found that three elements added to the duration and LED variables had a higher discriminative ability in classifying the cognitive status in PD groups, which implied that these factors might contribute substantially to the prediction of cognitive deterioration. The above prediction models were cross‐validated using the same dataset divided into two sets: training and testing. After the assessment, the model performance was proved to be robust. We established an ROC model to classify PD and cognitive status employing the serum GDNF and degree centrality of frontal and parietal lobes that exhibited the performance of features that had an acceptable accuracy. The degree centrality combined with serum GDNF ROC model based on voxels features might promote the individualized diagnosis of PD and cognition dysfunction.

Brain networks are fundamental for cognitive function. Previous research has indicated that functional properties can be exerted by brain regions without the need for direct physical or structural connections. Furthermore, dynamic changes in functional networks can lead to a reshaping of the physical structure of brain networks through plasticity. This raises the question of how changes in connectivity at the biological or anatomical level. Specifically, can changes in the connectivity of regional structures be reflected at the anatomical level through the plasticity of cortical thickness? We aim to investigate whether changes in the connectivity of the frontal and parietal lobes correspond to changes in cortical thickness. Therefore, we further characterized cortical thickness to determine changes in the whole brain structure in our participants according to the above analysis regarding the degree of centrality difference. As a whole, in the PD group, there was a significant thinning of the cortical thickness involving frontal and temporal regions. Our results are supported by previous studies that found a frontal and temporal cortical thickness reduction or atrophy in PD patients with mild cognitive impairment.^37^, ^38^ Furthermore, correlation analysis of the cognitive results and cortical thickness subdomain showed that the left medial frontal gyrus thickness involves multiple cognitive functions, including orientation, memory, attention, and execution. The middle frontal gyrus has been reported as the cortical focus for both the storage of semantic memory and the processing components of working memory in the human brain.^39^, ^40^ BA46, also included in this area, is primarily involved in the sustained mnemonic response.^39^ We suggest that their reduced connectivity and decreased thickness indicate a decline in cognition, particularly in respect of spatial locations, memory, and language.^39^, ^41^

Additionally, activation in the left middle frontal cortex (L‐MFC) is involved in a task that requires executive attention. The thickness of the L‐MFC has shown a statistically significant positive correlation with organizational attention performance.^42^ Indeed, other studies have clarified that structural deficits in the MFC, such as thickness reduction, could lead to multiple abnormalities in regulating emotion and cognition in PD.^43^ Therefore, L‐MFC thickness evaluation is significant in respect of the judgment of disease and the severity of cognitive impairment. As a result, we propose that the reduction in L‐MFC thickness alone could be a marker to diagnose PD. Combining serum GDNF levels, PD duration, and education levels could be used to distinguish the degree of cognitive impairment.

The current study has some limitations. First, the numbers of patients were relatively small. However, despite the small sample size, we were able to identify the brain network connectivity and cortical thickness alterations due to the minimum sample size we forecast at G‐power is guaranteed. Second, all PD participants undertook an fMRI scan with no suspension of their daily medication. Some studies have emphasized that in subjects in the dopamine medication “off” state, global and local efficiency are decreased in the brain network topology properties.^44^, ^45^ A broader patient sample should be used, particularly in respect of drug‐naive individuals. However, medicine was examined as a covariable factor in our study. The association between treatment and alterations in imaging should also be focused on and explored in future research. Third, another limitation of the study is its cross‐sectional design. According to our findings, it is possible to assume a gradual decrease in cortical thickness, but given that the decrease in cortical thickness increases with the duration and severity of the disease, this requires confirmation of a longitudinal sample. Fourth, in the absence of pathological confirmation and extra follow‐up datasets, the current data cannot establish the pathological mechanism of early cognitive decline in brain abnormalities of some regions and cortical thickness. In the future, it is still necessary to verify the ROC classification model through multiple datasets and establish a dynamic relationship between GDNF levels and cognition status. Last, multiple algorithmic segmentation models are deserved to be investigated in the future to clarify the specificity and accuracy of different algorithms in revealing certain aspects of neuroimaging.

On the whole, we found that PD patients with different GDNF level not only have abnormal cerebral cortical morphological changes, but also have abnormal topological properties changes at the level of large‐scale structural networks. Besides, cognitive abnormalities in PD were associated with degree centrality and cortical thickness alterations. These changes may be the potential pathophysiological mechanism of PD clinical manifestations. It has certain guiding significance for PD clinical diagnosis, individual treatment, and rehabilitation exercise. Combining serum GDNF with these imaging indicators (such as the degree centrality of cluster1 and cluster2, and the cortical thickness of the left middle frontal cortex) may achieve higher sensitivity and specificity or be more accurate for disease or cognitive dysfunction diagnosis compared to a single indicator. Although our results need confirmation in further studies, they show that a simple algorithm combining age, education, as well as serum GDNF and imaging parameters can classify cognitive status, in the appropriate clinical context, clinicians and researchers can use the proposed method to evaluate the degree of cognition objectively and calculate risk of cognitive decline for individuals with early Parkinson's disease.

## AUTHOR CONTRIBUTIONS

Chuanxi Tang and Dianshuai Gao conceived the project and designed the study (Conceptualization); Chuanxi Tang, Mingyu Sh, Ruiao Sun, and Ke Xue wrote the manuscript (Writing); Sijie Liang, Gang Chen, Mingyu Sh, and Changyu Wu performed clinical peripheral blood samples collection and cognitive evaluation, Imaging scan (Investigation, Methodology); Piniel Alphayo and Dianshuai Gao provided scientific input and English‐editing work; Chuanxi Tang, Mengying Wang, Ruiao Sun, and Gang Chen contributed to analysis(formal analysis, validation, visualization).

## CONFLICT OF INTEREST STATEMENT

The authors declare no competing interests.

## Supporting information


Figure S1

Table S1

## Data Availability

The authors declare that the primary data supporting the findings of this study are available within the article and its Supplementary Information files. Additional data are available from the corresponding author upon request.

## References

[1] Armstrong MJ , Okun MS . Diagnosis and treatment of Parkinson disease: a review. JAMA. 2020;323(6):548‐560. doi:10.1001/jama.2019.22360 32044947

[2] Dorsey ER , Elbaz A , Nichols E , et al. Global, regional, and national burden of Parkinson's disease, 1990–2016: a systematic analysis for the global burden of disease study 2016. Lancet Neurol. 2018;17(11):939‐953. doi:10.1016/s1474-4422(18)30295-3 30287051 PMC6191528

[3] Bloem BR , Okun MS , Klein C . Parkinson's disease. Lancet. 2021;397(10291):2284‐2303. doi:10.1016/s0140-6736(21)00218-x 33848468

[4] Yarnall AJ , Breen DP , Duncan GW , et al. Characterizing mild cognitive impairment in incident Parkinson disease: the ICICLE‐PD study. Neurology. 2014;82(4):308‐316. doi:10.1212/WNL.0000000000000066 24363137 PMC3929202

[5] Pont‐Sunyer C , Hotter A , Gaig C , et al. The onset of nonmotor symptoms in Parkinson's disease (the ONSET PD study). Mov Disord. 2015;30(2):229‐237. doi:10.1002/mds.26077 25449044

[6] Emre M . Clinical features, pathophysiology and treatment of dementia associated with Parkinson's disease. Handb Clin Neurol. 2007;83:401‐419. doi:10.1016/S0072-9752(07)83018-1 18808925

[7] Aarsland D , Batzu L , Halliday GM , et al. Parkinson disease‐associated cognitive impairment. Nat Rev Dis Primers. 2021;7(1):47. doi:10.1038/s41572-021-00280-3 34210995

[8] Delgado‐Alvarado M , Gago B , Navalpotro‐Gomez I , Jimenez‐Urbieta H , Rodriguez‐Oroz MC . Biomarkers for dementia and mild cognitive impairment in Parkinson's disease. Mov Disord. 2016;31(6):861‐881. doi:10.1002/mds.26662 27193487

[9] Fereshtehnejad SM , Zeighami Y , Dagher A , Postuma RB . Clinical criteria for subtyping Parkinson's disease: biomarkers and longitudinal progression. Brain. 2017;140(7):1959‐1976. doi:10.1093/brain/awx118 28549077

[10] van den Heuvel MP , Hulshoff Pol HE . Exploring the brain network: a review on resting‐state fMRI functional connectivity. Eur Neuropsychopharmacol. 2010;20(8):519‐534. doi:10.1016/j.euroneuro.2010.03.008 20471808

[11] Sizemore AE , Phillips‐Cremins JE , Ghrist R , Bassett DS . The importance of the whole: topological data analysis for the network neuroscientist. Netw Neurosci. 2019;3(3):656‐673. doi:10.1162/netn_a_00073 31410372 PMC6663305

[12] Mu L , Zhou Q , Sun D , Wang M , Chai X , Wang M . The application of resting magnetic resonance imaging in the cognitive judgment of Parkinson. World Neurosurg. 2020;138:672‐679. doi:10.1016/j.wneu.2020.02.002 32545020

[13] Petrou M , Bohnen NI , Muller ML , Koeppe RA , Albin RL , Frey KA . Abeta‐amyloid deposition in patients with Parkinson disease at risk for development of dementia. Neurology. 2012;79(11):1161‐1167. doi:10.1212/WNL.0b013e3182698d4a 22933741 PMC3525303

[14] Marks W , Evers L , Faber M , Verbeek M , de Vries N , Bloem B . Study design for a multi‐modal approach to understanding Parkinson's disease: the personalized Parkinson project. Movement Disord. 2017;32(suppl2). https://www.mdsabstracts.org/abstract/study‐design‐for‐a‐multi‐modal‐approach‐to‐understanding‐parkinsons‐disease‐the‐personalized‐parkinson‐project/

[15] Tome D , Fonseca CP , Campos FL , Baltazar G . Role of neurotrophic factors in Parkinson's disease. Curr Pharm Des. 2017;23(5):809‐838. doi:10.2174/1381612822666161208120422 27928963

[16] Liu Y , Tong S , Ding L , Liu N , Gao D . Serum levels of glial cell line‐derived neurotrophic factor and multiple neurotransmitters: in relation to cognitive performance in Parkinson's disease with mild cognitive impairment. Int J Geriatr Psychiatry. 2020;35(2):153‐162. doi:10.1002/gps.5222 31650626

[17] Shi MY , Ma CC , Chen FF , et al. Possible role of glial cell line‐derived neurotrophic factor for predicting cognitive impairment in Parkinson's disease: a case‐control study. Neural Regen Res. 2021;16(5):885‐892. doi:10.4103/1673-5374.297091 33229724 PMC8178776

[18] Chauhan NB , Siegel GJ , Lee JM . Depletion of glial cell line‐derived neurotrophic factor in substantia nigra neurons of Parkinson's disease brain. J Chem Neuroanat. 2001;21(4):277‐288. doi:10.1016/s0891-0618(01)00115-6 11429269

[19] Deinhardt K , Chao MV . Trk receptors. Handb Exp Pharmacol. 2014;220:103‐119. doi:10.1007/978-3-642-45106-5_5 24668471

[20] Tang CX , Chen J , Shao KQ , et al. Blunt dopamine transmission due to decreased GDNF in the PFC evokes cognitive impairment in Parkinson's disease. Neural Regen Res. 2023;18(5):1107‐1117. doi:10.4103/1673-5374.355816 36255000 PMC9827775

[21] Carmichael K , Sullivan B , Lopez E , Sun L , Cai H . Diverse midbrain dopaminergic neuron subtypes and implications for complex clinical symptoms of Parkinson's disease. Ageing Neurodegener Dis. 2021;1(4). doi:10.20517/and.2021.07 PMC8442626 34532720

[22] Samantaray T , Saini J , Gupta CN . Sparsity dependent metrics depict alteration of brain network connectivity in Parkinson's disease. Annu Int Conf IEEE Eng Med Biol Soc. 2022;2022:698‐701. doi:10.1109/EMBC48229.2022.9871258 36085972

[23] Wang Z , Yuan Y , Bai F , You J , Zhang Z . Altered topological patterns of brain networks in remitted late‐onset depression: a resting‐state fMRI study. J Clin Psychiatry. 2016;77(1):123‐130. doi:10.4088/JCP.14m09344 26845269

[24] Liu J , Li M , Pan Y , et al. Complex brain network analysis and its applications to brain disorders: a survey. Complexity. 2017;2017:1‐27. doi:10.1155/2017/8362741

[25] Shindy WW , Posley KA , Fuster JM . Reversible deficit in haptic delay tasks from cooling prefrontal cortex. Cereb Cortex. 1994;4(4):443‐450. doi:10.1093/cercor/4.4.443 7950314

[26] Herbet G , Moritz‐Gasser S , Duffau H . Electrical stimulation of the dorsolateral prefrontal cortex impairs semantic cognition. Neurology. 2018;90(12):e1077‐e1084. doi:10.1212/WNL.0000000000005174 29444964

[27] Gigi A , Babai R , Penker A , Hendler T , Korczyn AD . Prefrontal compensatory mechanism may enable normal semantic memory performance in mild cognitive impairment (MCI). J Neuroimaging. 2010;20(2):163‐168. doi:10.1111/j.1552-6569.2009.00386.x 19490403

[28] Liu H , Deng B , Zhou H , et al. QEEG indices are associated with inflammatory and metabolic risk factors in Parkinson's disease dementia: an observational study. EClinicalMed. 2022;52:101615. doi:10.1016/j.eclinm.2022.101615 PMC9399166 36034410

[29] Thota N , Lenka A , George L , et al. Impaired frontal lobe functions in patients with Parkinson's disease and psychosis. Asian J Psychiatr. 2017;30:192‐195. doi:10.1016/j.ajp.2017.10.013 29101795

[30] Duncan J . The structure of cognition: attentional episodes in mind and brain. Neuron. 2013;80(1):35‐50. doi:10.1016/j.neuron.2013.09.015 24094101 PMC3791406

[31] Premji A , Zapallow C , Tsang P , Tang R , Jacobs M , Nelson AJ . Influence of area 5 on interhemispheric inhibition. Neuroreport. 2011;22(18):974‐978. doi:10.1097/WNR.0b013e32834d8806 22027515

[32] Whitlock JR . Posterior parietal cortex. Curr Biol. 2017;27(14):R691‐R695. doi:10.1016/j.cub.2017.06.007 28743011

[33] Igelstrom KM , Graziano MSA . The inferior parietal lobule and temporoparietal junction: a network perspective. Neuropsychologia. 2017;105:70‐83. doi:10.1016/j.neuropsychologia.2017.01.001 28057458

[34] Compta Y , Parkkinen L , O'Sullivan SS , et al. Lewy‐ and Alzheimer‐type pathologies in Parkinson's disease dementia: which is more important? Brain. 2011;134(Pt 5):1493‐1505. doi:10.1093/brain/awr031 21596773 PMC4194668

[35] Zhou M , Yang C , Bu X , et al. Abnormal functional network centrality in drug‐naive boys with attention‐deficit/hyperactivity disorder. Eur Child Adolesc Psychiatry. 2019;28(10):1321‐1328. doi:10.1007/s00787-019-01297-6 30798413

[36] Wang Y , Yu N , Lu J , et al. Increased effective connectivity of the left parietal lobe during walking tasks in Parkinson's disease. J Parkinsons Dis. 2023;13(2):165‐178. doi:10.3233/JPD-223564 36872789 PMC10041419

[37] Hanganu A , Bedetti C , Jubault T , et al. Mild cognitive impairment in patients with Parkinson's disease is associated with increased cortical degeneration. Mov Disord. 2013;28(10):1360‐1369. doi:10.1002/mds.25541 23801590

[38] Chung SJ , Yoo HS , Lee YH , et al. Frontal atrophy as a marker for dementia conversion in Parkinson's disease with mild cognitive impairment. Hum Brain Mapp. 2019;40(13):3784‐3794. doi:10.1002/hbm.24631 31090134 PMC6865684

[39] Leung HC , Gore JC , Goldman‐Rakic PS . Sustained mnemonic response in the human middle frontal gyrus during on‐line storage of spatial memoranda. J Cogn Neurosci. 2002;14(4):659‐671. doi:10.1162/08989290260045882 12126506

[40] Gabrieli JD , Poldrack RA , Desmond JE . The role of left prefrontal cortex in language and memory. Proc Natl Acad Sci USA. 1998;95(3):906‐913. doi:10.1073/pnas.95.3.906 9448258 PMC33815

[41] Hazem SR , Awan M , Lavrador JP , et al. Middle frontal gyrus and area 55b: perioperative mapping and language outcomes. Front Neurol. 2021;12:646075. doi:10.3389/fneur.2021.646075 33776898 PMC7988187

[42] Andersson M , Ystad M , Lundervold A , Lundervold AJ . Correlations between measures of executive attention and cortical thickness of left posterior middle frontal gyrus ‐ a dichotic listening study. Behav Brain Funct. 2009;5:41. doi:10.1186/1744-9081-5-41 19796388 PMC2761925

[43] Asami T , Takaishi M , Nakamura R , et al. Cortical thickness reductions in the middle frontal cortex in patients with panic disorder. J Affect Disord. 2018;240:199‐202. doi:10.1016/j.jad.2018.07.064 30077161

[44] Achard S , Bullmore E . Efficiency and cost of economical brain functional networks. PLoS Comput Biol. 2007;3(2):e17. doi:10.1371/journal.pcbi.0030017 17274684 PMC1794324

[45] Skidmore F , Korenkevych D , Liu Y , He G , Bullmore E , Pardalos PM . Connectivity brain networks based on wavelet correlation analysis in Parkinson fMRI data. Neurosci Lett. 2011;499(1):47‐51. doi:10.1016/j.neulet.2011.05.030 21624430

