# Supplementary figures and images for: Distinct serum GDNF coupling with brain structural and functional changes underlies cognitive status in Parkinson's disease
Source: CNS Neurosci Ther. 2023 Sep 17;30(3):e14461. doi: 10.1111/cns.14461 (PMC10916445; doi:10.1111/cns.14461)

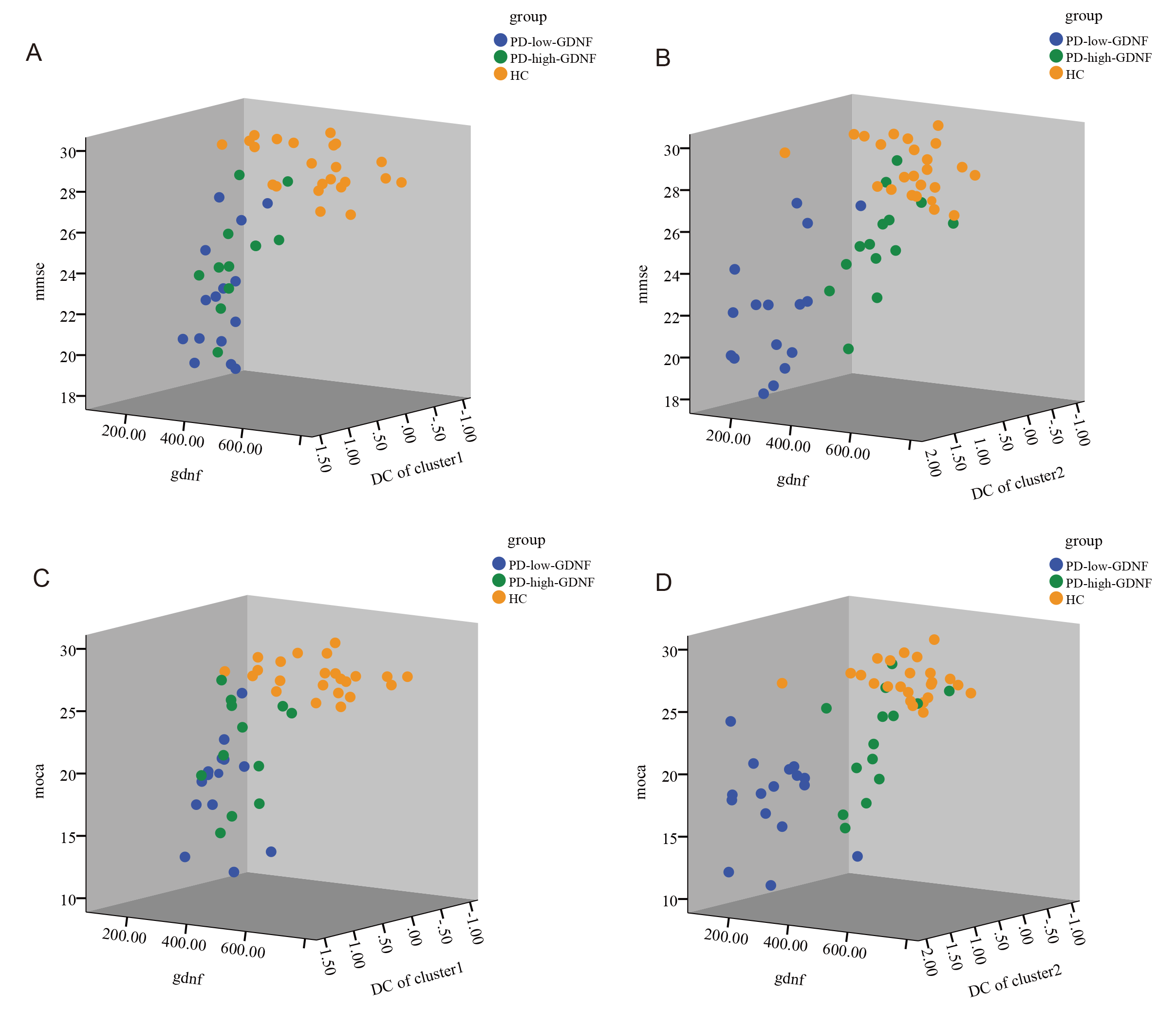

Supplement: Supplementary file 1 — Figure S1 [file CNS-30-e14461-s002.tif]
