# Supplementary material for: Distinct serum GDNF coupling with brain structural and functional changes underlies cognitive status in Parkinson's disease
Source: CNS Neurosci Ther. 2023 Sep 17;30(3):e14461. doi: 10.1111/cns.14461 (PMC10916445; doi:10.1111/cns.14461)
Supplement: Supplementary file 2 — Table S1 [file CNS-30-e14461-s001.docx]

**Supplementary Table1. Pearson’s correlation coefficients between degree centrality of voxel-wise clusters and cognition score, GDNF level in all subjects or PD cohort.**
